# Liquor Chlorinæ

**Published:** 1864-12

**Authors:** 


*Liquor Chlorinæ.*—Put four grains of chlorate of potash, well pulverized in an eight ounce bottle; then add half a drachm of hydrochloric acid; cork the bottle well, and let it remain for about five minutes; then add gradually eight ounces of water, shaking the bottle constantly until the whole of the water be added. Dose, from an ounce to a drachm. This probably does not contain enough of the chlorate to be of much service. The following formula has been largely and successfully used: chlorate of potash, two scruples; hydrochloric acid, thirty minims; tincture of opium, ten minims to one drachm; syrup, half an ounce; water, to six ounces. One drachm to one ounce for a dose. The opium prevents diarrhœa which frequently occurs during the administration of the chlorine mixture. For external application: chlorate of potash, one drachm; and one ounce each of the hydrochloric acid and water. Two drachms of this mixture to one pint of water makes an excellent detergent and stimulant application to wounds,

